# Optimised chronic infection models demonstrate that siderophore ‘cheating’ in *Pseudomonas aeruginosa* is context specific

**DOI:** 10.1038/ismej.2017.103

**Published:** 2017-07-11

**Authors:** Freya Harrison, Alan McNally, Ana C da Silva, Stephan Heeb, Stephen P Diggle

**Affiliations:** 1 School of Life Sciences, Gibbet Hill Campus, University of Warwick, Coventry, UK; 2 Centre for Biomolecular Sciences, School of Life Sciences, University of Nottingham, Nottingham, UK; 3 Institute of Microbiology and Infection, College of Medical and Dental Sciences, University of Birmingham, Birmingham, UK

## Abstract

The potential for siderophore mutants of *Pseudomonas aeruginosa* to attenuate virulence during infection, and the possibility of exploiting this for clinical ends, have attracted much discussion. This has largely been based on the results of *in vitro* experiments conducted in iron-limited growth medium, in which siderophore mutants act as social ‘cheats:’ increasing in frequency at the expense of the wild type to result in low-productivity, low-virulence populations dominated by mutants. We show that insights from *in vitro* experiments cannot necessarily be transferred to infection contexts. First, most published experiments use an undefined siderophore mutant. Whole-genome sequencing of this strain revealed a range of mutations affecting phenotypes other than siderophore production. Second, iron-limited medium provides a very different environment from that encountered in chronic infections. We conducted cheating assays using defined siderophore deletion mutants, in conditions designed to model infected fluids and tissue in cystic fibrosis lung infection and non-healing wounds. Depending on the environment, siderophore loss led to cheating, simple fitness defects, or no fitness effect at all. Our results show that it is crucial to develop defined *in vitro* models in order to predict whether siderophores are social, cheatable and suitable for clinical exploitation in specific infection contexts.

## Introduction

Bacteria can be social organisms, displaying coordinated behaviours, including quorum sensing (QS), biofilm formation and the production of diffusible exoproducts, which can be shareable ‘public goods’ (West *et al.*, 2007). Iron-scavenging siderophores, exoproteases, biofilm polymers, toxins and QS signals all have the potential to be public goods because the ability of a cell to benefit from their presence (take up a siderophore or the products of extracellular proteolysis) are not contingent on that cell having secreted the molecule itself. Cells which do not produce molecules that enhance growth or survival grow poorly or not at all in monoculture. However, in co-culture with wild-type cells, they avoid the metabolic costs of exoproduct synthesis and reap the benefits of their neighbours’ investment. Their evolutionary fitness relative to the wild-type increases and these mutants are commonly referred to as ‘cheats’ (Rainey and Rainey, 2003; Griffin *et al.*, 2004; Harrison *et al.*, 2006; Diggle *et al.*, 2007; Jiricny *et al.*, 2010; Darch *et al.*, 2012; Raymond *et al.*, 2012; Mund *et al.*, 2017). Circumstances under which the benefits of cheating are constrained, allowing cooperation to persist in populations, have been predicted by theory (Hamilton, 1964; Székely *et al.*, 2010). Because bacteria are amenable to evolution experiments, they have been widely used to test theoretical predictions *in vitro* (for example, Griffin *et al.*, 2004; Diggle *et al.*, 2007; Kümmerli *et al.*, 2009a). Siderophore production by fluorescent Pseudomonas is a particularly tractable workhorse for sociomicrobiology and has served as a useful model system to facilitate tests of key evolutionary hypotheses (Table 1, also Morgan *et al.*, 2012; Zhang and Rainey, 2013; Luján *et al.*, 2015).

Many exoproducts that could be considered public goods play important roles in infection. As the field of sociomicrobiology progressed, this led to suggestions that cooperation among pathogens could be usefully manipulated, especially in hard-to-treat chronic infections (for example, Foster, 2005; Harrison *et al.*, 2006; Brown *et al.*, 2009; Rumbaugh *et al.*, 2009). The opportunistic pathogen *Pseudomonas aeruginosa* causes various chronic infections in immunocompromised hosts, notably lung infections in people with cystic fibrosis (CF) and wound infections in people with burns or diabetic ulcers (Hirsch and Tam, 2010; Friedrich *et al.*, 2015). Siderophore-null mutants of this species can invade *in vitro* populations and cheat their way to high density, resulting in population reduction (Griffin *et al.*, 2004; Table 1). Siderophore-negative mutants are commonly isolated from chronic *P. aeruginosa* infections (De Vos *et al.*, 2001; Jiricny *et al.*, 2014; Andersen *et al.*, 2015). Researchers have therefore hypothesised that the presence of these mutants is due to cheating *in vivo*, and that siderophore mutants could be used as ‘Trojan horses’ to ferry antibiotic-susceptibility alleles into infections and render them more sensitive to treatment (Harrison *et al.*, 2006; Brown *et al.*, 2009; Jiricny *et al.*, 2014; Andersen *et al.*, 2015).

A potential problem with this reasoning is the implicit assumption that gene expression, the accessibility of exoproducts to non-producers and the fitness consequences of mutations are comparable *in vitro* and *in vivo*, and in acute and chronic infection contexts. Technical advances have revealed how much bacterial transcriptomes (Croucher and Thomson, 2010; Dötsch *et al.*, 2015) and the fitness of loss-of-function mutants (Van Opijnen *et al.*, 2009) vary across environments. When the environment that bacteria experience in a specific infection context is carefully modelled in the lab, the importance of this in determining the fitness consequences of a particular genotype become clear (Palmer *et al.*, 2007; Harrison *et al.*, 2014; Turner *et al.*, 2015).

Typically, experiments on siderophore cooperation and cheating are conducted in iron-limited minimal medium, creating clear costs and benefits to siderophore production and allowing null mutants to be cheats. While this may recapitulate the iron restriction encountered by pathogens colonising healthy tissues during acute infection, chronic infections present a very different environment. Tissue damage, inflammation and disease-specific changes in-host phenotype (for example, increased mucus volume and adhesiveness in CF lungs, Boucher, 2007) are accompanied by changes in the growth substrates available to bacteria. In CF lungs, bacteria use amino acids released by damaged tissues, or from mucus, as carbon sources (Palmer *et al.*, 2005, 2007; Flynn *et al.*, 2016), and iron is plentiful (Tyrrell and Callaghan, 2016). Consequently, bacterial gene expression, and presumably the roles played by virulence factors, differ in chronic and acute contexts (Goodman *et al.*, 2004). More specifically, in CF, there is evidence that *P. aeruginosa* switches from siderophore-mediated uptake of iron (III) to utilising available iron (II) and heme (Cornelis and Dingemans, 2013; Marvig *et al.*, 2014). Further, experiments have typically used well-mixed liquid medium where exoproducts and cells move freely. Adding an element of spatial structure to the *in vitro* environment locally restricts the accessibility of siderophores, rendering them less ‘public’ and reducing the ability of non-producing mutants to cheat (Kümmerli *et al.*, 2009c; Luján *et al.*, 2015; Leinweber *et al.*, 2017). Because chronic infections caused by bacterial biofilm in host tissues are structured (Bjarnsholt, 2013), similar restrictions may apply *in vivo*. Clearly, there is a pressing need to re-assess the role played by siderophore mutants in environments that better model chronic infection. This is now possible because lab models of the environments encountered in CF lungs and soft-tissue wounds have been developed (Palmer *et al.*, 2007; Werthén *et al.*, 2010; Harrison *et al.*, 2014; Turner *et al.*, 2015; Harrison and Diggle, 2016).

To assess and extend the potential of laboratory experiments to yield clinically useful data on the evolutionary dynamics of *P. aeruginosa* siderophore mutants, we reviewed published experimental work. Our goal was to characterise any biases in the literature which could restrict its applicability to the various environments in which this flexible opportunist can thrive. We found two such biases: (a) many experiments (by ourselves and others) used an uncharacterised, UV-generated mutant (PAO6609, often referred to as PAO9) as a siderophore cheat; and (b) a lack of studies employing a well-defined model of chronic infection. We defined two empirical aims to address these biases.

First, we conducted whole-genome sequencing of PAO6609 to determine the genetic basis of its siderophore-null phenotype, and reveal any other mutations that could affect the outcome of competition with the wild type. Second, we determined the fitness consequences of siderophore loss-of-function mutations in relatively well-characterised models of CF lung infection and chronic soft-tissue wounds. Because we found many mutations that potentially affect metabolism and growth in PAO6609, we used defined siderophore deletion mutants of the siderophores pyoverdine and pyochelin for these experiments. We explicitly explored differences between liquid chronic infection medium and structured infection models because spatial structure affects the social dynamics of public goods (Kümmerli *et al.*, 2009c; Luján *et al.*, 2015; Leinweber *et al.*, 2017; Mund *et al.*, 2017), and because biofilm formation in structured models of lung (Harrison and Diggle, 2016) and wound (Werthén *et al.*, 2010) infection could alter gene expression and the fitness consequences of mutation (Whiteley *et al.*, 2001; Bjarnsholt *et al.*, 2013). Our results demonstrate that whether siderophore mutants act as cheats is dependent on the environment, and suggest that these mutants may not act as cheats *in vivo*.

## Materials and methods

### Bacterial strains

*P. aeruginosa* PAO1 was used as a wild-type siderophore producer and defined Δ*pvdD* and Δ*pvdD*Δ*pchEF* mutants in this background (Ghysels *et al.*, 2004) were used as single pyoverdine and double pyoverdine/pyochelin mutants, respectively. Recent literature shows that PAO1 strains fall into two discrete phenotypic groups termed P1 and P2, based on presence or absence of indels in the *mexT* locus (Luong *et al.*, 2014). We Sanger sequenced the *mexT* locus of these strains and aligned it against the University of Washington PAO1 genome (NCBI reference sequence NC_002516), which has an 8 bp insertion in *mexT* producing the P2 phenotype. None of our strains had this insertion, nor did they have a commonly-reported 2 bp deletion present in other P2 strains (Luong *et al.*, 2014). Thus our strains have the P1 phenotype (data not shown). The UV-induced mutant PA6609 was derived from PAO6049 (Hohnadel *et al.*, 1986); PAO6049 is a Tn5-induced methionine auxotroph derived from PAO1 (Rella *et al.*, 1985). See Supplementary Figure S1 for the genealogy of PAO6609.

### Collating and analysing experimental work on siderophore cheating

To identify published experiments on siderophore cheating, we used Scopus.com to (i) conduct a literature search and (ii) locate articles citing already-identified experimental and theoretical articles on siderophore social evolution. For each experiment in each article, we recorded which strains/clones and media were used; whether an explicit test for cooperation was conducted (relative fitness of mutant in pure/mixed culture with a producer strain and growth or final density of pure/mixed cultures); the starting frequency of mutants in co-culture experiments; the location of the key data in the article; whether cheating was observed and any other key conclusions. The diverse starting frequencies and mutants used make this data unsuitable for formal meta-analysis, so we simply present the key characteristics and findings of the studies in a format that facilitates qualitative comparison.

### Whole-genome sequencing of PAO6609 (PAO9)

PAO6609 was cultured overnight in 10 ml Lysogeny Broth at 37 °C on an orbital shaker. Genomic DNA was extracted using a Sigma-Aldrich (Gillingham, UK) GenElute Bacterial Genomic DNA Kit. Library preparation was performed using the Nextera XT library preparation kit (Illumina Inc., San Diego, CA, USA), and 2 × 300 bp paired-end sequencing performed on the Illumina MiSeq platform using a V3 sequencing cartridge, to ~50 × coverage. Reads were assembled using SPAdes run with the –careful flag, and annotated using Prokka. This produced an assembly of 6 232 039 bp comprising 89 contigs with an N50 of 220 789. The assembly was compared with the reference PAO1 genome (NCBI reference sequence NC_002516) using BLAST and the comparison visualised using ACT to search for gene acquisition or loss events. SNP typing was performed by mapping the raw reads of PAO6609 against the PAO1 reference genome (NC_002516.2) using SMALT and SAMtools. A total of 98.3% of 1.17M reads mapped to the reference, from which high fidelity SNPs were called using a cutoff of minimum allele frequency of 0.8, minimum quality score 30, and minimum depth of 8.

### Growth conditions for cheating experiments

We used five different growth environments. In each case, cultures were grown in 24-well microtitre plates and incubated on a rocking platform at 37 °C. (i) 2 ml casamino acids medium (CAA: 5 g casamino acids, 1.18 g K_2_HPO_4_·3H_2_O, 0.25 g MgSO_4_·7H_2_O, per litre) supplemented with 20 mm NaHCO_3_ and 100 μg ml^−1^ human apo-transferrin (Sigma, Gillingham, UK), cultured for 24 h. (ii) 2 ml artificial sputum medium (ASM) following recipe in Palmer *et al.* (2007); cultured for 24 or 48 h. (iii) *ex vivo* pig lung (EVPL) model comprising 5 mm^2^ pig bronchiole+400 μl ASM following protocol in Harrison and Diggle (2016); cultured for 96 h. (iv) 2 ml synthetic wound fluid (SWF: 50% v/v peptone water/foetal bovine serum following Werthén *et al.* (2010)), cultured for 24 or 48 h. (v) 400 μl synthetic chronic wound (SCW: SWF solidified with rat tail collagen following protocol in Werthén *et al.* (2010)), cultured for 48 h. For environments (ii–v), we measured wild-type siderophore production and calculated the fitness of siderophore mutants relative to the wild type in clonal and mixed culture. Four or five replica cultures were inoculated with (a) wild-type, (b) Δ*pvdD* (c) Δ*pvdD*Δ*pchEF*, (d) 50% wild-type+50% Δ*pvdD* or (e) 50% wild-type+50% Δ*pvdD*Δ*pchEF* bacteria for each environment/culture time combination, and each experiment was repeated twice to yield two experimental blocks. For environment i (CAA), we simply measured siderophore production by four replica populations of the wild type for comparison with environments (ii-v).

The density of the inoculum was kept as consistent as possible across environments and population types, as cell density can affect the outcome of wild-type—siderophore-null co-culture experiments (Ross-Gillespie *et al.*, 2009). For experiments in CAA, ASM and SWF, each culture was inoculated with a total of two colonies of the relevant genotype(s), picked from LB plates using a 200 μl pipette tip. For experiments in EVPL and SCW, each culture was inoculated with two colonies of the relevant genotype(s), picked from LB plates using an insulin syringe fitted with a 30 G needle.

To construct time courses of bacterial growth in ASM, two colonies of (a) wild-type, (b) Δ*pvdD* or (c) Δ*pvdD*Δ*pchEF* were inoculated into four 2 ml aliquots of ASM in a 24-well microtitre plate. This was incubated at 37 °C in a Tecan Infinite 200 Pro multimode reader for 18 h; every 20 min, the plate was shaken for 2 s and absorbance of each well read at 400 nm. (Reading at more traditional values of 600–700 nm risks interference from absorbance by pyoverdine in wild-type cultures).

### Calculation of relative fitness

The fitness of mutants relative to the wild type in mixed cultures, *v*, was calculated as *x*_2_(1-*x*_1_)/*x*_1_(1-*x*_2_), where *x*_1_ is the starting frequency of the mutant and *x*_2_ is the end frequency. To calculate the relative fitness of mutants in pure culture, mutant populations were randomly paired with pure wild-type cultures from the same experimental block and the same formula used. It follows from the definition that *v*<1 reflects a decrease in mutant frequency over the period of competition, that is, the mutant is less fit than the wild type, while *v*>1 reflects an increase in mutant frequency, that is, the mutant is fitter than the wild type. If mutant and wild type have the same fitness, then *v*=1.

### Statistical analyses

The data were analysed in R 3.3.0 (R Development Core Team, 2016) using general linear models and ANOVA. To meet model assumptions of homoscedasticity and normality of residuals, the fitness data from SWF were log-transformed and fitness data from ASM and EVPL square-root transformed; the fitness data from SCWs did not require transformation. All data on pyoverdine production, pyochelin production and growth for wild-type monocultures in the different growth environments were log transformed to meet model assumptions. Where missing values caused non-orthogonality, the *car* package (Fox and Weisberg, 2011) was used to perform ANOVA with Type II sums of squares, returning correct F-ratios for main effects in models containing an interaction. Fitted means and confidence intervals were retrieved, and post *hoc* contrasts tested, using the 'lsmeans' package, with default correction of multiple *P-*values for false discovery rate (Lenth, 2013). Post *hoc* multiple comparisons against a control were implemented using the 'glht' command in the 'multcomp' package (Hothorn *et al.*, 2008).

### Data availability

**Figure 1:**
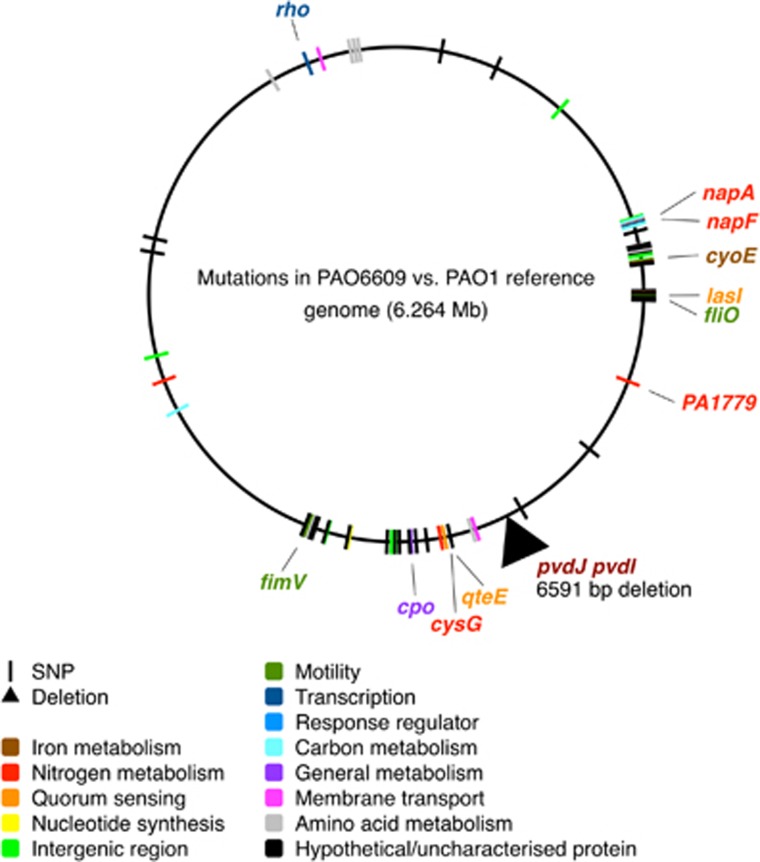
Location of one deletion and 90 SNPs in the genome of PAO6609 (a.k.a. PAO9), mapped against a PAO1 reference sequence (NC_002516.2). SNPs are colour coded by functional class of the locus affected. 11 SNPs that that result in amino acid substitution and which seem most likely to affect growth and virulence are highlighted and the gene name given.

**Figure 2:**
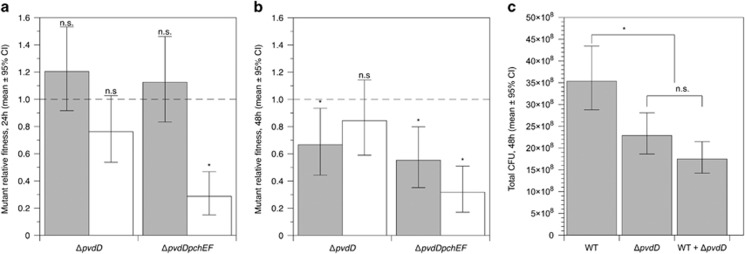
(**a**, **b**) Relative fitness of Δ*pvdD* and Δ*pvdD*Δ*pchEF* mutants in pure culture (grey bars) and in mixed culture with an isogenic wild type (white bars) in ASM after (**a**) 24 and (**b**) 48 h of growth. After eliminating variation due to experimental block, we found significant effects on 24-h fitness of mutant genotype (ANOVA: F_1,33_=6.96, *P*=0.012), presence/absence of the wild type (F_1,33_=26.8, *P*<0.001) and their interaction (F_1,33_=4.49, *P*<0.042). Neither mutant had a relative fitness significantly different from 1 when grown in pure culture (post *hoc t-*tests, *P*⩾0.17). Δ*pvdD* mutant fitness was unaffected by the wild type (relative fitness in mixed culture not significantly different from 1; post *hoc t*-test, *P*=0.074), but the Δ*pvdD*Δ*pchEF* mutant was outcompeted in mixed culture (post *hoc t*-test, *P*<0.001). The only significant determinant of 48-h fitnes was genotype (ANOVA eliminating experimental block: genotype F_1,35_=8.34, *P*=0.007; presence/absence of the wild type: F_1,33_=0.277, *P*=0.602; interaction term: F_1,33_=3.62, *P*=0.065). Post *hoc t*-tests showed that the Δ*pvdD* mutant was less fit than the wild type when grown in pure culture (*P*=0.018) but as fit as the wild type in mixed culture (*P*=0.279). The Δ*pvdD*Δ*pchEF* mutant had a relative fitness <1 in both pure and mixed culture (*P*⩽0.001). (**c**) Cheating by Δ*pvdD* over 48 h of co-culture results in mixed wild-type+mutant cultures showing the same reduction in total population density as pure mutant cultures (ANOVA eliminating experimental block, F_2,26_=12.6, *P*<0.001; Dunnett’s test for mutant/mixed populations versus wild type: *P*=0.009 and <0.001, respectively; post *hoc t-*test for mutant versus mixed populations, *P*=0.159). In all panels, bars show means of 9–10 replicates split across two replica experiments, with associated 95% confidence interval. In both panels, results of post *hoc* tests for relative fitness=1 are shown with *=*P*<0.02; n.s.=not significant.

**Figure 3:**
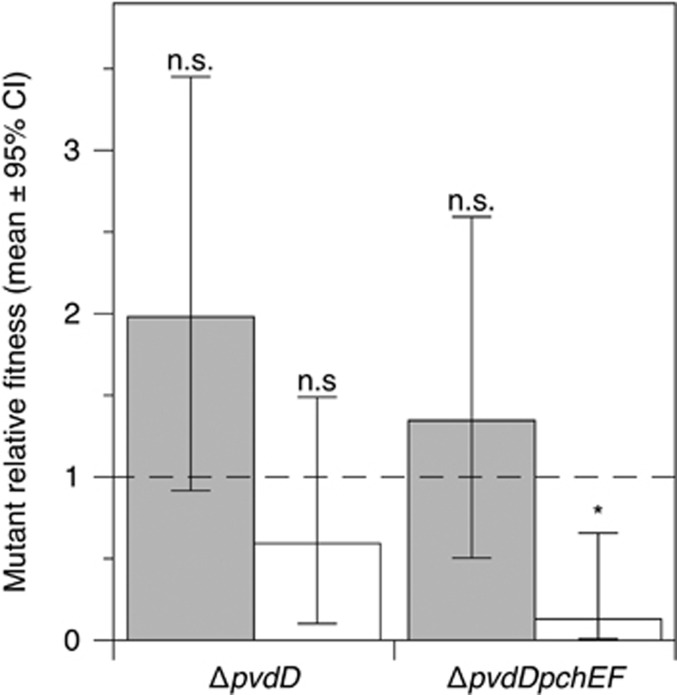
Relative fitness of Δ*pvdD* and Δ*pvdD*Δ*pchEF* mutants in pure culture (grey bars) and in mixed culture with an isogenic wild type (white bars) in *ex vivo* pig lung+ASM after 96 h of growth. Bars show means of eight replicates spread across two replica experiments, with associated 95% confidence interval. (ANOVA eliminating experimental block: genotype F_1,27_=2.24, *P*=0.146; presence/absence of the wild type: F_1,27_=10.7, *P*=0.003; interaction term: F_1,27_=0.137, *P*=0.714). Neither strain had a relative fitness signficantly different from 1 in pure culture (post *hoc t-*tests, *P*=0.074 for Δ*pvdD*, *P*=0.472 for Δ*pvdD*Δ*pchEF*). In co-culture, relative fitness was not significantly different from 1 for Δ*pvdD* (*P*=0.074), but <1 for Δ*pvdD*Δ*pchEF* (*P*=0.007). In both panels, results of post *hoc* tests for relative fitness=1 are shown with *=*P*=0.007; n.s.=not significant.

**Figure 4:**
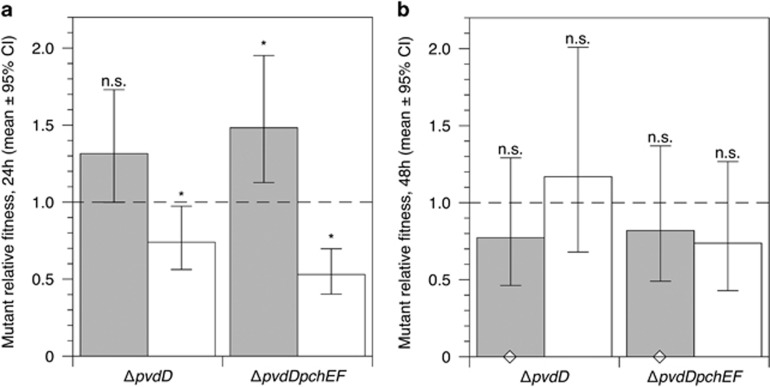
Relative fitness of Δ*pvdD* and Δ*pvdD*Δ*pchEF* mutants in pure culture (grey bars) and in mixed culture with an isogenic wild type (white bars) in SWF after (**a**) 24 and (**b**) 48 h of growth. Bars show means of 10 replicates split across two replica experiments, with associated 95% confidence interval. Twenty-four hour fitnss was detemrined only by the presence/absnece of the wild type (ANOVA eliminating experimental block: genotype F_1,35_=0.621, *P*=0.436; presence/absence of wild type: F_1,35_=35.2, *P*<0.001; interaction term: F_1,35_=2.82, *P*=0.102); The Δ*pvdD* mutant’s relative fitness was not significantly different from 1 in pure culture (post *hoc t*-test, *P*=0.051), and the Δ*pvdD*Δ*pchEF* mutant slightly and significantly >1 (*P*=0.006), but both were less fit than the wild type in mixed culture (*P*=0.032 and <0.001, respectively). Forty-eight-hour fitness was not affected by genotype or co-culture (ANOVA eliminating experimental block: genotype: F_1,35_=0.045, *P*=0.833; presence/absence of wild type: F_1,35_=1.34, *P*=0.255; interaction term: F_1,35_=0.078, *P*=0.781): both mutants had a relative fitness of 1 regardless of wild type presence (post *hoc t-*tests, *P*⩾0.104). In both panels, results of post *hoc* tests for relative fitness=1 are shown with *=*P*<0.02; n.s.=not significant. Confidence intervals and *P-*values in (**b**) are taken from a model that excluded two outliers where relative fitness was zero (indicated by open diamonds): including these outliers in the analysis did not change any of the conclusions given in the text.

**Figure 5:**
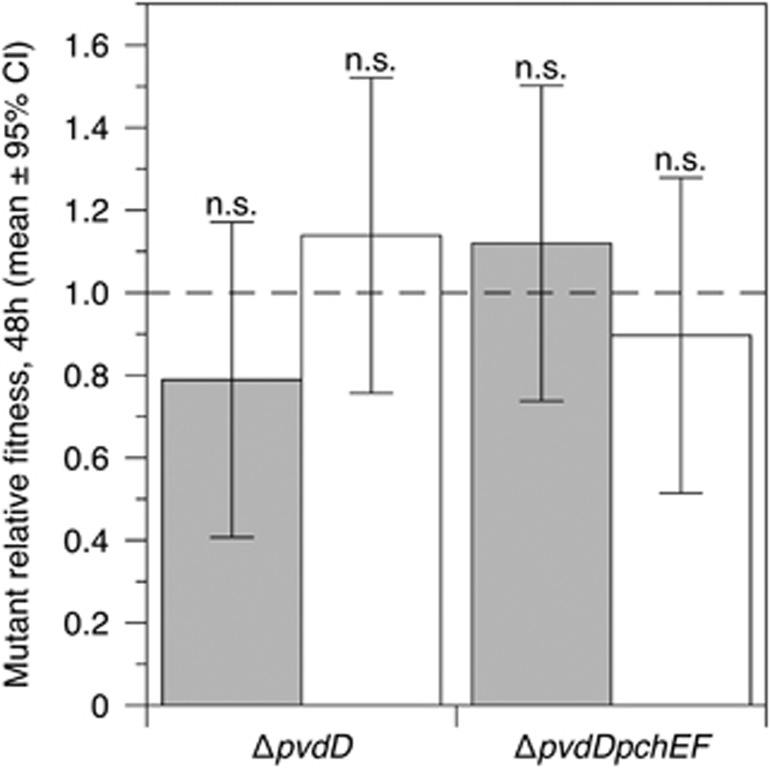
Relative fitness of Δ*pvdD* and Δ*pvdD*Δ*pchEF* mutants in pure culture (grey bars) and in mixed culture with an isogenic wild type (white bars) in synthetic wounds after 48 h of growth. Relative fitness was unaffected by genotype or culture condition (ANOVA eliminating experimental block: genotype F_1,35_=0.055, *P*=0.817; presence/absence of wild type: F_1,35_=0.113, *P*=0.739; interaction term: F_1,35_=2.32, *P*=0.137; post *hoc t*-tests for relative fitness=1, *P*⩾0.270). Bars show means of 10 replicates split across two replica experiments, with associated 95% confidence interval and result of post *hoc* tests (all n.s.).

The raw sequence data has been deposited in the European Nucleotide Archive (Accession number: ERR1725797). Summary information on mutations in PAO6609 (Figure 1) and the raw data for experiments depicted in Figures 2, 3, 4, 5,Supplementary Figure S2 are available as Supplementary Information at the *ISME Journal* website.

## Results

### Defining key research priorities: analysis of published experiments.

A review of the published experiments on *P. aeruginosa* siderophore mutants and cheating identified 36 experiments in 23 published articles: these are summarised in Table 1. It should be noted that the motivations behind these experiments were diverse, and generally sought to understand the evolutionary dynamics of siderophores in iron-limited environments without any direct reference to potential clinical relevance. Nevertheless, we can use these experiments to gain a picture of the state of the art in siderophore research and assess how far this could inform understanding of chronic infection. Two-thirds of the experiments used the well-characterised lab strain PAO1 as the siderophore-producing wild type, and six experiments used clinical or environmental isolates. Of the 35 experiments that did not focus on natural isolates, 15 used defined siderophore mutants. These included deletions of the *pvdD/pvdF* and/or *pchEF* loci, which are involved in the biosynthesis of the siderophores pyoverdine and pyochelin, respectively. Of the remainder, 10 used the UV-induced mutant PAO6609 (Supplementary Figure S1) and five used spontaneous or evolved mutants.

We recorded whether each experiment included a specific test for cheating. We determined a ‘cheating’ mutant to be a non-producer which (1) had lower fitness (grew less well) than the producer genotype in monoculture, and (2) gained a growth advantage in mixed culture, such that it grew as well or better than the wild type, that is, the mutant was maintained in the population and may have increased in frequency. Thus, we defined a specific test for cheating as a quantitative comparison of the growth or fitness of producers and non-producers in monoculture and co-culture, and recorded the location of the data pertaining to this test in each publication.

Out of 36, 34 experiments in Table 1 were conducted in CAA medium or minimal salts supplemented with CAA as a carbon source; in all but two cases, the media was made more iron-limited by the addition of the human iron chelator transferrin: one study did not use apotransferrin at all and one explicitly compared CAA with and without apotransferrin. This medium was not designed to reflect any specific natural environment: it was optimised to ensure that siderophores were necessary for growth, and acted as a public good, when bacteria were cultured in it (Griffin *et al.*, 2004). This makes it ideal for experiments to test evolutionary theory about why and how different social strategies can be selected for. Experiments in iron-limited CAA have, for instance, revealed that the potential for siderophore mutants to cheat can be reduced or curtailed by the scale of competition in a metapopulation (Griffin *et al.*, 2004) or by growth in a structured environment, where spatial segregation prevents mutants from accessing siderophores produced by wild types (Kümmerli *et al*, 2009c; Leinweber *et al.*, 2017). As an opportunist with a large, flexible metabolome and secretome, *P. aeruginosa* can persist in a range of environments. Some of these may be approximated by iron-limited minimal media. For instance, experiments conducted in CAA may therefore be useful for understanding the dynamics of siderophore genotypes/phenotypes at the onset of acute infection in healthy host tissues, where iron is sequestered by high-affinity host chelators (including transferrin), or in nutrient-poor abiotic environments.

Four studies explicitly tested the effect of environmental variables on siderophore production in monoculture. Leinweber *et al.* (2017) showed that the ability of a double pyoverdine/pyochelin mutant to cheat in CAA is curtailed when the medium is not made strictly iron limited with apotransferrin. Brockhurst *et al.* (2008) showed that increasing resource supply (carbohydrates and amino acids) reduced the cost of siderophore production and so reduced the cheating benefits of siderophore loss. Dumas *et al.* (2013) dissected the relative roles of pyoverdine and pyochelin, suggesting that while pyoverdine is the primary siderophore expressed under severe iron limitation, under moderate iron limitation pyochelin production dominates. The authors used parameters estimated from their experiments to simulate wild-type—siderophore mutant competition in a range of environmental scenarios and found that the fitness consequences of siderophore-null mutations varied. On the basis of these results, the authors suggested that pyoverdine-deficient mutants may be favoured in chronic infection due to better growth in high-iron, low-pH environments, rather than because they are cheats. The importance of environmental iron regime and pyoverdine/pyochelin switching in determining the fitness consequences of siderophore mutants has also been explored in a suite of experiments using the closely-related bacterium *P. fluorescens*: pyoverdine mutants of this species were shown to cheat under severe, but not moderate, iron limitation (Zhang and Rainey, 2013; Kümmerli and Ross-Gillespie, 2014). Finally, Vasse *et al.* (2017) showed that the presence of an antibiotic could enhance the fitness benefits accruing to a pyoverdine mutant in co-culture with the wild type.

Further, the strength of siderophore cheating may be constrained. Cheating could become self-limiting as increases in mutant frequency alter the cost:benefit ratio of losing siderophore production, or the cost:benefit ratio may be different in actively-growing versus established populations. In many studies, in Table 1 that explored a range of starting frequencies, mutants act as cheats only when they are initially inoculated at frequencies⩽0.1, which raises questions about the ability of these mutants to cheat at and persist at higher frequencies (although a few studies did report cheating from very high starting frequencies—for example, Kümmerli *et al.*, 2009b; Leinweber *et al.*, 2017). In some cases, even though co-culture increased mutant relative fitness to>1, there was no detrimental effect on total population density. These observations call into question the ability of siderophore mutants to act as ‘Trojan horses’ to treat infection, as does the observation in one study (Ghoul *et al.*, 2016) that a siderophore mutant only cheats if added to wild-type cultures before stationary phase.

Therefore, whether siderophore-null mutants act as cheats in chronic infection—and why apparently comparable experiments can give different results—remains an open question. We identified two key areas that require close attention in order to gain a more realistic picture of the consequences of siderophore null mutations in chronic infections.

First, the repeated use of PAO6609 is potentially problematic. This is a UV-induced mutant derived from PAO6049 (Hohnadel *et al.*, 1986), which is a methionine auxotroph derived from PAO1 (Rella *et al.*, 1985) following several deliberate and spontaneous mutational events. We have reconstructed the complex genealogy of PAO6609 and provide this information as a flow chart in Supplementary Figure S1. The exact nature of the mutation leading to the siderophore-negative phenotype in PAO6609 is not known. Further, as UV mutagenesis is non-specific, it is likely that this strain carries additional mutations that influence other important aspects of its phenotype, or moderate the fitness consequences of siderophore loss. Without knowing the exact genotype of PAO6609, we cannot be sure that empirical results obtained using it are the result of its siderophore phenotype alone.

Second, while some of the studies in Table 1 explicitly addressed how environmental variables affect siderophore dynamics, none of them set out to model conditions relevant to chronic infection. It is possible that increased availability of iron in chronically-damaged tissues means that the benefits of siderophore production are reduced and that production is downregulated, removing any advantage to siderophore-null genotypes. Alternatively, siderophores may be beneficial but not exploitable by potential cheats: this could result from spatial structuring, persistence in stationary phase, and/or a reliance on siderophore recycling rather than continued production (Kümmerli and Brown, 2010).

### Whole-genome sequence of a commonly used siderophore cheat, PAO6609

We performed whole-genome sequencing of this strain and mapped the raw sequence data against a PAO1 reference genome (NCBI Reference Sequence: NC_002516.2, Stover *et al.*, 2000). The results are summarised in Figure 1. PAO6609 harbours a single ~6.6 kb deletion, removing most of the non-ribosomal peptide synthetase locus *pvdJ*, which is involved in synthesising the pyoverdine side chain. This deletion also removes part of a second pyoverdine biosynthetic locus, *pvdI*. We also found 90 high-resolution SNPs, including expected mutations in *amiE* and *metZ* (Supplementary Figure S1). The details of the deletion and SNPS are provided as Supplementary Information. Briefly, none of the SNPs were in loci associated with pyoverdine or pyochelin biosynthesis. However, we found SNPs resulting in missense mutations in ten genes. These were *cyoE*, which is involved in heme metabolism and upregulated when iron is low (Arai, 2011); two loci, which ancide an assimilatory nitrate reductase involved in anaerobic ATP generation (*napA*, *napF*); two loci involved with assimilation of nitrogen for biosynthesis (*cysG*, PA1779); two loci involved in quorum sensing (*lasI*, *qteE*); two loci involved in motility (*fliO*, *fimV*); and the *cpo* gene, which is involved in the biosynthesis of many organochlorine compounds. All of these mutations could potentially affect growth and/or virulence. Finally, we found a missense mutation in the *rho* transcription termination factor. Further work would be needed to deduce the effects of this mutation, but the possibility of generalised defects in the control of gene expression cannot be ruled out. In summary, PAO6609 harbours numerous mutations in addition to the *pvdJ* partial deletion that likely make it an unreliable strain to use for empirical studies of the fitness and virulence consequences of siderophore loss.

### Production of siderophores by *P. aeruginosa* in models of chronic lung and wound infections

Because of the problems identified with PAO6609, we used defined siderophore mutants to test the potential for social cheating in laboratory media that have been developed to represent specific chronic infection contexts. As discussed above, while most research has focused on pyoverdine, under weaker iron limitation, the metabolically cheaper siderophore pyochelin may take on a primary role in iron chelation (Dumas *et al.*, 2013; Kümmerli and Ross-Gillespie, 2014). We therefore used a standard wild-type lab strain (PAO1) and two isogenic deletion mutants: a pyoverdine knockout (Δ*pvdD*) and a double pyoverdine/pyochelin knockout (Δ*pvdD*Δ*pchEF*) constructed using allelic exchange (Ghysels *et al.*, 2004). Both mutants have been reported to act as social cheats in iron-limited CAA, when inoculated in co-culture with the wild type at starting frequencies of up to 50% (Table 1,Kümmerli *et al.*, 2009b, 2015; Kümmerli and Brown, 2010).

We first verified that PAO1 produces siderophores in our chronic infection models, and compared production levels with those in iron-limited CAA. Detectable levels of pyoverdine and pyochelin were produced in all environments (Supplementary Figure S2a). Cell densities also differed between environments (Supplementary Figure S2b). Therefore, we calculated relative production of siderophores per CFU and used this measure of production to compare investment between the different growth environments. Per-cell levels of pyoverdine and pyochelin were positively correlated and, consistent with the suggestion of Dumas *et al.* (2013); the pyochelin:pyoverdine ratio was significantly higher in chronic infection models and media than in CAA (Supplementary Figure S2a). (Note that replica cultures in *ex vivo* lung and synthetic wounds were more variable than replica cultures in liquid media).

### Social dynamics of siderophore mutants in artificial CF sputum are influenced by genotype and culture time

Mutant and wild-type bacteria were grown in pure culture and each mutant was grown in mixed culture with the wild type, with a starting frequency of 50%, in ASM. After 24 and 48 h of growth, total population densities were determined and the relative fitness of each mutant in pure and mixed culture calculated. As shown in Figure 2a, neither mutant had a relative fitness significantly different from 1 when grown in pure culture. Δ*pvdD* mutant fitness was unaffected by the wild type, but the Δ*pvdD*Δ*pchEF* mutant was outcompeted in mixed culture. These results are not consistent with either mutant acting as a cheat: they do not show a disadvantage when cultured alone, and do not benefit from growth with the wild type.

After 48 h (Figure 2b), the results were different. The Δ*pvdD* mutant now showed cheating dynamics: it was less fit than the wild type when grown in pure culture but as fit as the wild type in mixed culture. The Δ*pvdD*Δ*pchEF* mutant had a relative fitness <1 in both pure and mixed culture. As predicted for a textbook ‘cheat,’ the presence of the Δ*pvdD* mutant reduced total population density: mixed cultures reached similar densities to pure Δ*pvdD* cultures (Figure 2c).

Time course experiments (Supplementary Figure S3) suggested that the Δ*pvdD* mutant has a disadvantage in pure culture in ASM because it ceases logarithmic growth earlier than the wild type, reaching a lower yield in stationary phase. The Δ*pvdD*Δ*pchEF* mutant has the double disadvantage of a longer lag phase and an earlier cessation of logarithmic growth. This is consistent with the differing fitness of the mutants at 24 vs 48 h: continued growth and siderophore production by the wild type from 24–48 h presumably provide an opportunity for the Δ*pvdD* mutant to cheat, as levels of available iron start to fall.

### Siderophore mutants are outcompeted by the wild type in a CF bronchiolar biofilm model

ASM models the chemistry of CF mucus (Palmer *et al.*, 2007; Turner *et al.*, 2015), but liquid culture lacks realistic spatial structure. To allow bacteria to form biofilms associated with bronchiolar surfaces (Bjarnsholt *et al.*, 2013), we repeated the monoculture and 50% co-culture experiments in an *ex vivo* model comprising a section of pig bronchiole cultured in ASM for 4 days. *P. aeruginosa* forms a loose sleeve of mucoid biofilm around the tissue (Harrison and Diggle, 2016). As shown in Figure 3, the only significant predictor of relative fitness was presence/absence of the wild type. Both strains were as fit as the wild type in pure culture but showed a trend towards being outcompeted in mixed culture. This trend was significant for the Δ*pvdD*Δ*pchEF* mutant but not for the Δ*pvdD* mutant.

### Siderophore mutants do not suffer a long-term fitness disadvantage in synthetic wound fluid

We next repeated the pure/mixed culture experiments in synthetic wound fluid (SWF: Werthén *et al.*, 2010). The only significant predictor of relative fitness at 24 h was presence/absence of the wild type (Figure 4a). The Δ*pvdD* mutant was as fit as the wild type in pure culture, and the Δ*pvdD*Δ*pchEF* mutant slightly and significantly fitter, but both mutants were outcompeted by the wild type in mixed culture. After 48 h (Figure 4b), there was no difference between genotypes or culture conditions: both mutants had a relative fitness of 1 regardless of wild-type presence. Dropping two outliers from the 48-h data set resulted in tighter confidence intervals but did not change the results.

### Siderophore mutants do not have any fitness disadvantage in synthetic chronic wounds

As with the CF lung model, we wished to add spatial structure to SWF to better model a soft tissue infection. SWF was solidified with collagen, allowing bacteria to form 3D biofilm, (Werthén *et al.*, 2010) and experiments repeated in the resulting solid plugs. After 48 h in synthetic wounds, (Figure 5), both mutants had a relative fitness of 1 regardless of culture condition.

## Discussion

Many experiments have been conducted to explore the social evolution of siderophore production by *P. aeruginosa.* These have been used to suggest explanations for the appearance of siderophore mutants in chronic infections; and how this behaviour could be exploited for clinical ends. Our targeted literature review revealed potential restrictions on the generality of predictions made from these experiments about the dynamics of siderophore mutants *in vivo*. First, many experiments used an undefined mutant (PA6609) as a siderophore cheat. We sequenced this strain and found that while it has a deletion of one of the pyoverdine biosynthetic loci, it also carries numerous mutations in genes likely to affect growth and metabolism in unpredictable ways. It is therefore difficult to disentangle the effects of siderophore phenotype vs other phenotypes on its evolutionary dynamics. Second, most published experiments were carried out in iron-limited minimal broth. Inferences made from these experiments may provide information on the fitness consequences of siderophore loss in acute infections (Granato *et al.*, 2016). However, a growing body of evidence shows a significant mismatch between contrived lab media and clinically-relevant growth conditions. In chronic infections, bacteria experience specialised and idiosyncratic environments that affect the cost:benefit ratio of siderophore production, the ability of cells to access each other’s siderophores and the growth rate of bacterial populations. Experiments that dissect the fitness consequences of siderophore loss in environmentally-explicit models of infection are essential if we are to understand the natural history of siderophore production in chronic infections. Defined chronic infection models are also vital if we want to draw clinically-relevant conclusions about the likely success of therapeutic interventions based on disrupting bacterial social interactions or using ‘public good’ mutants as Trojan horses.

We tested whether pyoverdine or pyoverdine/pyochelin deletion mutants acted as cheats in four laboratory models designed to mimic chronic infections: structured and unstructured models of CF lung infections and non-healing soft-tissue wounds. Wild-type *P. aeruginosa* produced siderophores in all tested environments. However, there was environment-dependent variation in siderophore production, and the ratio of pyochelin:pyoverdine was higher in chronic infection models than in CAA (Supplementary Figure S1). This is consistent with a suggestion by other authors that pyochelin production may be favoured when iron is more easily available (Dumas *et al.*, 2013; Hunter *et al.*, 2013; McCallin *et al.*, 2015). The pyoverdine mutant was less fit than the wild type in monoculture in artificial CF sputum after 48 h growth, demonstrating a benefit to pyoverdine production in this environment, and was able to cheat on the wild type. In all other environments tested, there was no effect of losing pyoverdine production on fitness. Production of pyoverdine in these environments may therefore be a maladaptive response, or may have benefits other than simple growth rate enhancements (for example, copper detoxification: Koh and Henderson, 2015). There was a non-significant trend towards this mutant being outcompeted by the wild type in *ex vivo* bronchiolar biofilm. This may be due to poor biofilm production, even when iron is plentiful (Banin *et al.*, 2005; Harrison and Buckling, 2009). The pyoverdine/pyochelin mutant was less fit than the wild type in artificial CF sputum and model bronchiolar biofilms, but had no disadvantage in synthetic wound fluid or synthetic wound biofilm. This is most likely due to ready availability of iron as heme groups in serum. Our results cast significant doubt on the ability of siderophore mutants to cheat in chronic infections, and therefore on their utility as Trojan horses.

The models we use are not perfect, but they have been carefully constructed to reflect key aspects of CF lung or wound physiology and chemistry, and validated by comparing bacterial growth, gene expression, metabolism and/or biofilm morphology with qualitative or quantitative data from clinical fluids or tissues (Palmer *et al.*, 2007; Werthén *et al.*, 2010; Harrison and Diggle, 2016; Price *et al.*, 2016). Alternative optimised media are available (for example, other versions of synthetic CF sputum: Sriramulu *et al.*, 2005; Turner *et al.*, 2015 and Kirchner *et al.*, 2012), and *P. aeruginosa* can be grown in chronic infection-like media and models for long time periods, with different studies using various growth periods. We decided to focus on the four media presented, and conduct our experiments over the most commonly used time periods (based on our sampling of the literature) for each growth medium or model. Clearly, there are many variables to be considered with regard to modelling chronic infection environments, and we stress that the present article presents the data from a representative sample of potential models. We would not venture to argue that the conditions we use are the ‘correct’ ones: rather, we present our results as examples of how improved lab models of specific infection contexts can be developed and used, and to demonstrate how different the fitness effects of a bacterial phenotype can be in different environments.

When increased levels of public goods production increase the population growth rate or carrying capacity, cheating mutants are predicted to be under negative frequency-dependent selection (Ross-Gillespie *et al.*, 2007). It is therefore usual to conduct cheating assays using a range of starting frequencies, including <50%. We chose to initially conduct cheating assays using only a 50% starting frequency, as both mutants we used have been reported to act as cheats under this condition in iron-limited CAA (Kümmerli *et al.*, 2009b, 2015; Kümmerli and Brown, 2010). Further, if mutants that cheat from low starting frequencies cannot maintain this advantage as they become more common, then the likely clinical significance of cheating as a determinant of virulence—and the potential power of a ‘Trojan horse’ approach to managing infection—is called into question. Further investigation of different starting frequencies was not necessary: in the case of ASM, the single mutant showed cheating dynamics even at this high starting frequency, and in all other media, both mutants had equal fitness to the wild type in pure culture so cannot be called cheats regardless of the outcome of competition at any starting frequency.

Our results underline the importance of the in-host environment in determining bacterial genotype-fitness maps, and hence the necessity of carefully-designed lab models (Roberts *et al.*, 2015). We previously reported that *P. aeruginosa* QS mutants, which can cheat in acute infections (Rumbaugh *et al.*, 2009), are not cheats in an *ex vivo* model of chronic infection (Harrison *et al.*, 2014). This is consistent with previously published *in vitro* experiments showing that the fitness effects of QS mutations depend on the specific carbon sources present (D’Argenio *et al.*, 2007; Ghoul *et al.*, 2014). Together with the present results, this demonstrates the unreliability of extrapolating predictions about bacterial social evolution from one infection context to another, or from simple lab media to specialised *in vivo* environments. In chronic lung and wound biofilm infections, availability of free iron, weakly-chelated iron or heme most likely reduces the benefit of producing siderophores and so production levels (Supplementary Figure S2). If siderophores are less important for growth, and/or reduced production and strong spatial structure in tissue- or mucus-embedded biofilms restrict siderophore availability, then clinical strategies to reduce bacterial load based on manipulating siderophore social interactions will not work.

So why do siderophore-null mutants arise during chronic infection? Are they cheats, or simply better adapted to local growth conditions? Are they not selected at all, but present transiently and/or at low frequencies? We cannot answer these questions with the current data.

Careful choice and optimisation of *in vitro* models that allow for long-term evolution experiments in realistic environments will be invaluable in providing answers. Alongside such models, there is also a need for quantitative, rather than qualitative, data from patients on the prevalence of siderophore mutants (see Mowat *et al.*, 2011 for an example of a quantitative approach to studying haplotype turnover in CF). There is also a need for more considered choice of ‘wild type’ and mutant genotypes, paying particular attention to genotypes that commonly arise during chronic infection. The fitness consequences of siderophore loss could depend on the nature of the mutations involved, and on background genotype. In future, it would be useful to conduct experiments with a range of clinical isolates that are most typical of those seen in patients, and/or with constructed mutants that recapitulate these. Further, future experiments using chronic infection models should also consider the impact of inter-species interactions in polymicrobial chronic infections communities: *P. aeruginosa* is part of a diverse microbial flora in CF lung and chronic wound biofilms, and interactions with co-infecting microbes have the potential to affect the costs and benefits of siderophore production (for example, Weaver and Kolter, 2004; Harrison *et al.*, 2008).

For instance, we and other authors have focussed on experiments with mutants with impaired siderophore biosynthesis. However, a recent study of siderophore mutants isolated from CF patients found that the majority carried mutations in the regulatory gene *pvdS* (Andersen *et al.*, 2015). Sequencing clones from an *in vitro* evolution experiment also revealed pyoverdine-negative phenotypes that stemmed from *pvdS* mutation (Kümmerli *et al.*, 2015). *PvdS* is a sigma factor that positively regulates the expression of pyoverdine biosynthesis under iron starvation (Miyazaki *et al.*, 1995), but which also positively regulates the expression of other virulence-related exoproducts (Ochsner *et al.*, 1996; Wilson *et al.*, 2001; Hunt *et al.*, 2002; Gaines *et al.*, 2007).

Longitudinal sputum sampling from CF patients (Andersen *et al.*, 2015) showed that clones with mutations that reduce or ablate pyoverdine production (mainly in *pvdS*) initially retain receptors for iron-loaded pyoverdine, but gain mutations in the receptor once pyoverdine-producing clones disappear from the sampled populations. This was interpreted by the authors as evidence for cheating driving the loss of siderophores. But in the absence of explicit tests for cheating by these isolates in co-culture with co-isolated wild types, using growth media that mimics lung biofilm, the longitudinal pattern presented does not unequivocally prove this conclusion. Other possible explanations include early selection for loss of *pvdS* due to other downstream phenotypes and/or reduced transcriptional costs; a relaxation of purifying selection for functional siderophore receptors in the late stages of infection when iron is plentiful; a bias in the data set towards early isolates, which increases the conditional probability that later isolates are also siderophore-null; or a combination of these factors.

Our results demonstrate the necessity of evaluating and modelling in-host environments as carefully as possible if we aim to understand in-host microbiology. Lab experiments in simple *in vitro* conditions have limited ecological and clinical validity, and so limited predictive power. A plethora of data on the chemical and microbial ecology of chronic infection is now available, providing abundant material for researchers wishing to study the natural history of pathogens in the lab (for example, for CF: Yang *et al.*, 2011; Folkesson *et al.*, 2012; Quinn *et al.*, 2014; Cowley *et al.*, 2015; Kyle *et al.*, 2015; Roberts *et al.*, 2015; Turner *et al.*, 2015; Flynn *et al.*, 2016). This information is ripe for consideration by microbiologists who justifiably see great potential for manipulating the in-host ecology of pathogens in order to halt the progression of debilitating chronic infection.

## Figures and Tables

**Table 1:** Results of a review of the empirical literature on *P. aeruginosa* siderophore mutants and cheating

*Reference*	*Producer strain*	*Non-producer strain*	*Growth medium*	*Specific test for cooperation*	*Location of results in publication*	*Non-producer start frequency*	*Cheating observed?*	*Notes*
Griffin *et al.*, 2004	ATCC 15692 (PAO1)	PA06609 (PAO9)	CAA+apotransferrin	Yes	Text	0.5	Variable	Cheating was concluded on the basis of differences in density of pure vs mixed culture, relative fitness not reported.
Harrison *et al.*, 2006	ATCC 15692 (PAO1)	PA06609 (PAO9)	Waxworm	Yes	Figures 3 and 4	0.03–0.9	No	Highest reported cheat relative fitness is 1, at start frequencies⩽0.01
Ross-Gillespie *et al.*, 2007	ATCC 15692 (PAO1)	PA06609 (PAO9)	CAA+apotransferrin	Yes	Figure 3	0.001–0.99	Variable	Cheating observed at starting frequencies⩽0.1; total population density only affected at lowest start frequency
Ross-Gillespie *et al.*, 2007	ATCC 15692 (PAO1)	PAO1 Δ*pvdD*	CAA+apotransferrin	Yes	Figure 3	0.001–0.99	Variable	Cheating observed at starting frequencies of 0.001, but no effect on total population density. This mutant gains less fitness benefit from co-culture than PAO9 does
Ross-Gillespie *et al.*, 2007	UCBPP-PA14 (clinical)	Spontaneous mutant	CAA+apotransferrin	Yes	Figure 3	0.001–0.99	Variable	Cheating observed at start frequencies⩽0.1, but no effects on total population density. This mutant gains less fitness benefit from co-culture than PAO9 does
Harrison *et al.*, 2008	PAO985	*De novo* evolution experiment	CAA+apotransferrin/iron,±*S. aureus*	No	Figures 1 and 2	0		
Brockhurst *et al.*, 2008	ATCC 15692 (PAO1)	PA06609 (PAO9)	M9 minimal salts+CAA+apotransferrin	No, but raw data available	Figure 4 & raw data	0.5	Yes	Cheat relative fitness in mixed culture decreases as more resources are supplied to the media (CAA concentration manipulated to alter carbohydrate and amino acid supply)
Kümmerli *et al.*, 2009a	ATCC 15692 (PAO1)	PA06609 (PAO9)	CAA+apotransferrin	Yes	Text	0.33	Yes	
Kümmerli *et al.*, 2009b	ATCC 15692 (PAO1)	PAO1 Δ*pvdD*	CAA+apotransferrin	No				
Kümmerli *et al.*, 2009b	ATCC 15692 (PAO1)	PAO1 Δ*pvdD*/Δ*pchEF*	CAA+apotransferrin	Yes	Figure 4	0.17–0.83	Yes	Cheat is always fitter than the wild type, even when competed at a starting frequency of 0.83.
Kümmerli *et al.*, 2009c	ATCC 15692 (PAO1)	PA06609 (PAO9)	CAA+apotransferrin	Yes	Figure 2	0.33	Variable	Cheating is time dependent
Harrison and Buckling, 2009	ATCC 15692 (PAO1)	*pvdF* transposon mutant in MPAO1 background (PA2396-C04::ISlacZ/hah)	CAA+apotransferrin	No, but raw data available	Raw data	0.5	No	Re-analysis of raw data reveals this mutant is less fit then the wild type in mixtures with a starting frequency of 0.5 in planktonic and biofilm culture.
Harrison and Buckling, 2009	ATCC 15692 (PAO1)	Clones evolved from PAO6049	CAA+apotransferrin	No	Raw data	0.5	No	Mutants outcompete the wild type in planktonic mixed culture, but so does their siderophore-pruducing ancestor & they are lab adapted, growing as well as PAO1 in pure culture.
Ross-Gillespie *et al.*, 2009	ATCC 15692 (PAO1)	PA06609 (PAO9)	CAA+apotransferrin	Yes	Figure 2	0.09	No	
Ross-Gillespie *et al.*, 2009	ATCC 15692 (PAO1)	PAO1 Δ*pvdD*	CAA+apotransferrin	Yes	Figure 2	0.09	Variable	Cheating observed at high cell density only.
Ross-Gillespie *et al.*, 2009	UCBPP-PA14 (clinical isolate)	Spontaneous mutant	CAA+apotransferrin	Yes	Figure 2	0.09	Variable	Cheating observed at high cell density only.
Kümmerli *et al.*, 2010	ATCC 15692 (PAO1)	PA06609 (PAO9)	CAA+apotransferrin	Yes	Text	0.5	Yes	
Kümmerli and Brown, 2010	ATCC 15692 (PAO1)	PAO1 Δ*pvdD*	CAA+apotransferrin	Yes	Figure 5	0.5	Yes	
Kümmerli and Brown, 2010	PAO6049	PA06609 (PAO9)	CAA+apotransferrin	No				
Kümmerli and Brown, 2010	Environmental isolate	Spontaneous mutant	CAA+apotransferrin	Yes	Figure 5	0.5	Yes	
Jiricny *et al.*, 2010	ATCC 15692 (PAO1)	PAO1 Δ*pvdD*	CAA+apotransferrin	Yes	Figures 4 and 5	0.09	Yes	
Jiricny *et al.*, 2010	ATCC 15692 (PAO1)	PAO1 Δ*pchEF*	CAA+apotransferrin	No		0.09		
Jiricny *et al.*, 2010	ATCC 15692 (PAO1)	PAO1 Δ*pvdD*/Δ*pchEF*	CAA+apotransferrin	No		0.09		
Jiricny *et al.*, 2010	11 various isolates	Spontaneous mutants	CAA+apotransferrin	Yes	Figures 4 and 5	0.09	Variable	Cheating observed for 8/11mutants. Three non-cheats (with high pyoverdin production) may be driving the reported trend.
Harrison and Buckling, 2011	PAO6049	Evolved clones	CAA+apotransferrin	Yes	Figure 1	0.05, 0.5	Variable	Cheating observed at low starting frequencies for a minority of mutants (mean relative fitness=1)
Harrison and Buckling, 2011	PAO1 Δ*mutS*	Evolved clones	CAA+apotransferrin	Yes	Figure 1	0.05, 0.5	Variable	Cheating observed at low starting frequencies only for a majority of mutants, but some can cheat from a starting frequency of 0.5.
Dumas and Kümmerli, 2012	ATCC 15692 (PAO1)	Evolved clones	CAA+apotransferrin	No	Figures 3 and 4	0		Pyoverdine-deficient clones evolved, but their presence did not consistently reduce population growth—some were associated with increased growth.
Dumas *et al.*, 2013	ATCC 15692 (PAO1)	PAO1 Δ*pvdD* and Δ*pvdD*/Δ*pchEF*	CAA+apotransferrin	No	Figure 4	Three-strain mix, each strain at 0.33		Carbon source, pH and temperature determine the relative growth advantage conferred by siderophores in monoculture. In acidic pH, Δ*pvdD* grows better. Report outcome of simuated competitions based on monoculture growth parameters.
Ghoul *et al.*, 2014	Cystic fibrosis isolate	2x spontaneous mutants	CAA	Yes	Figures 3 and 4	0.1	Yes	Mutant with reduced pyoverdine production cheats on parent strain, and is cheated on by a second spontaneous mutant with even lower pyoverdine production.
Ross-Gillespie *et al.*, 2015	PAO1 Δ*pvdD*	PAO1 Δ*pvdD*/Δ*pchEF*	CAA+apotransferrin	Yes	Figure 2	0.2	Yes	This experiment competed a double pyoverdine/pyochelin knockouts against a single pyoverdine knockout.
Ross-Gillespie *et al.*, 2015	PAO1 Δ*pchEF*	PAO1 Δ*pvdD*/Δ*pchEF*	CAA+apotransferrin	Yes	Figure 2	0.2	Yes	This experiment competed a double pyoverdine/pyochelin knockouts against a single pyochelin knockout.
Kümmerli *et al.*, 2015	ATC 15692 (PAO1)	PAO1 Δ*pvdD*	CAA+apotransferrin	No, but can be inferred from data supplied	Figures 1,2,3	0.5	Yes	There is a coevolutionary arms race, whereby producers become less exploitable and non-producers become better cheats. Non-producer frequency is negatively correlated with population growth. Non-producers grow less well than producers in monoculture, and contemporary pairs show relative fitness of non-producers is >1 (though graphs suggest this effect is small).
Andersen *et al.*, 2015	Cystic fibrosis isolates	CF isolates	No culture	No	Text			Cheating inferred by sequence of mutations affecting pyoverdine production and uptake. Very few mutations reported in pyoverdine biosynthetic loci and none pyochelin loci. Most mutations are in pvdS.
Ghoul *et al.*, 2016	ATCC 15692 (PA01)	PA06609 (PAO9)	CAA+apotransferrin	Yes	Figures 1a and 2a	0.02–0.10	Variable	Mutant cheats only if added to producer cultures before the onset of stationary phase.
Leinweber *et al.*, 2017	ATCC 15692 (PA01)+eGFP tag	PAO1 Δ*pvdD*/Δ*pchEF*+mCherry tag.	CAA±apotransferrin	Yes	Figures 1,2c and 3,Supplementary Figure S1	0.1, 0.5, 0.9	Yes	Mutant was fitter than the wild type in shaken liquid medium (no spatial structure) and as fit as the wild type in static medium or medium solidified with agar (spatial structure present) when apotransferrin was added, regardless of starting frequency. No cheating observed when apotransferrin was not added.
Vasse *et al.*, 2017	ATCC 15692 (PA01)	PAO1 Δ*pvdD*	CAA+apotransferrin	Yes	Supplementary Figure S1 and Figure 1	0.15, 0.45, 0.75	Yes	When progressively higher concentrations of gentamicin are added to the medium, mutants lose their growth disadvanatge in monoculture but gain a larger benefit from co-culture with the wild type.

Abbreviation: CAA, casamino acids medium.

## References

[bib1] Andersen SB, Marvig RL, Molin S, Krogh Johansen H, Griffin AS. (2015). Long-term social dynamics drive loss of function in pathogenic bacteria. Proc Natl Acad Sci 112: 10756–10761. 26240352 10.1073/pnas.1508324112 PMC4553784

[bib2] Arai H. (2011). Regulation and function of versatile aerobic and anaerobic respiratory metabolism in *Pseudomonas aeruginosa*. Front Microbiol 2: 103. 21833336 10.3389/fmicb.2011.00103 PMC3153056

[bib3] Banin E, Vasil ML, Greenberg EP. (2005). Iron and *Pseudomonas aeruginosa* biofilm formation. Proc the Natl Acad Sci USA 102: 11076–11081. 10.1073/pnas.0504266102 PMC1182440 16043697

[bib4] Bjarnsholt T. (2013). The role of bacterial biofilms in chronic infections. APMIS 121: 1–58. 23635385 10.1111/apm.12099

[bib5] Bjarnsholt T, Alhede M, Alhede M, Eickhardt-Sørensen SR, Moser C, Kühl M et al. (2013). The *in vivo* biofilm. Trends Microbiol 21: 466–474. 23827084 10.1016/j.tim.2013.06.002

[bib6] Boucher RC. (2007). Airway surface dehydration in cystic fibrosis: pathogenesis and therapy. Annu Rev Med 58: 157–170. 17217330 10.1146/annurev.med.58.071905.105316

[bib7] Brockhurst MA, Buckling A, Racey DA, Gardner A. (2008). Resource supply and the evolution of public-goods cooperation in bacteria. BMC Biol 6: 20. 18479522 10.1186/1741-7007-6-20 PMC2409295

[bib8] Brown SP, West SA, Diggle SP, Griffin AS. (2009). Social evolution in micro-organisms and a Trojan horse approach to medical intervention strategies. PhilosTrans R Soc B Biol Sci 364: 3157–3168. 10.1098/rstb.2009.0055 PMC2781867 19805424

[bib9] Cornelis P, Dingemans J. (2013). *Pseudomonas aeruginosa* adapts its iron uptake strategies in function of the type of infections. Front Cell Infect Microbiol 14: 75. 10.3389/fcimb.2013.00075 PMC3827675 24294593

[bib10] Marvig RL, Damkiær S, Khademi SMH, Markussen TM, Molin S, Jelsbak L. (2014). Within-host evolution of *Pseudomonas aeruginosa* reveals adaptation toward iron acquisition from hemoglobin. mBio 5: e00966–14. 24803516 10.1128/mBio.00966-14 PMC4010824

[bib11] Cowley ES, Kopf SH, LaRiviere A, Ziebis W, Newman DK. (2015). Pediatric cystic fibrosis sputum can be chemically dynamic, anoxic, and extremely reduced due to hydrogen sulfide formation. mBio 6: e00767–15. 26220964 10.1128/mBio.00767-15 PMC4551978

[bib12] Croucher NJ, Thomson NR. (2010). Studying bacterial transcriptomes using RNA-seq. Curr Opin Microbiol 13: 619–624. 20888288 10.1016/j.mib.2010.09.009 PMC3025319

[bib13] D’Argenio DA, Wu M, Hoffman LR, Kulasekara HD, Déziel E, Smith EE et al. (2007). Growth phenotypes of *Pseudomonas aeruginosa lasR* mutants adapted to the airways of cystic fibrosis patients. Mol Microbiol 64: 512–533. 17493132 10.1111/j.1365-2958.2007.05678.x PMC2742308

[bib14] Darch SE, West SA, Winzer K, Diggle SP. (2012). Density-dependent fitness benefits in quorum-sensing bacterial populations. Proc Natl Acad Sci USA 109: 8259–8263. 22566647 10.1073/pnas.1118131109 PMC3361460

[bib15] De Vos D, De Chial M, Cochez C, Jansen S, Tummler B, Meyer JM, Cornelis P. (2001). Study of pyoverdine type and production by *Pseudomonas aeruginosa* isolated from cystic fibrosis patients: prevalence of type II pyoverdine isolates and accumulation of pyoverdine-negative mutations. Arch Microbiol 175: 384–388. 11409549 10.1007/s002030100278

[bib16] Diggle SP, Griffin AS, Campbell GS, West SA. (2007). Cooperation and conflict in quorum-sensing bacterial populations. Nature 450: 411–414. 18004383 10.1038/nature06279

[bib17] Dötsch A, Schniederjans M, Khaledi A, Hornischer K, Schulz S, Bielecka A et al. (2015). The *Pseudomonas aeruginos*a transcriptional landscape is shaped by environmental heterogeneity and genetic variation. mBio 6: e00749–00715. 26126853 10.1128/mBio.00749-15 PMC4488947

[bib18] Dumas Z, Kümmerli R. (2012). Cost of cooperation rules selection for cheats in bacterial metapopulations. J Evol Biol 25: 473–484. 22168669 10.1111/j.1420-9101.2011.02437.x

[bib19] Dumas Z, Ross-Gillespie A, Kümmerli R. (2013). Switching between apparently redundant iron-uptake mechanisms benefits bacteria in changeable environments. Proc R Soc B Biol Sci 280: 20131055. 10.1098/rspb.2013.1055 PMC3712426 23760867

[bib20] Flynn JM, Niccum D, Dunitz JM, Hunter RC. (2016). Evidence and role for bacterial mucin degradation in cystic fibrosis airway disease. PLoS Pathog 12: e1005846. 27548479 10.1371/journal.ppat.1005846 PMC4993466

[bib21] Folkesson A, Jelsbak L, Yang L, Johansen HK, Ciofu O, Hoiby N et al. (2012). Adaptation of *Pseudomonas aeruginosa* to the cystic fibrosis airway: An evolutionary perspective. Nat Rev Microbiol 10: 841–851. 23147702 10.1038/nrmicro2907

[bib22] Foster KR. (2005). Hamiltonian medicine: Why the social lives of pathogens matter. Science 308: 1269–1270. 15919984 10.1126/science.1108158

[bib23] Fox J, Weisberg S. (2011)An R Companion to Applied Regression. SAGE Publications: Thousand Oaks, CA, USA.

[bib24] Friedrich M, Lessnau K-D, Cunha BA. (2015), Pseudomonas aeruginosa Infections. In: Talavera F, Brusch JL (eds). Medscape Drugs and Diseases. Available at: http://emedicine.medscape.com/article/226748.

[bib25] Gaines JM, Carty NL, Tiburzi F, Davinic M, Visca P, Colmer-Hamood JA et al. (2007). Regulation of the *Pseudomonas aeruginosa toxA**regA* and *ptxR* genes by the iron-starvation sigma factor PvdS under reduced levels of oxygen. Microbiology 153: 4219–4233. 18048935 10.1099/mic.0.2007/011338-0

[bib26] Ghoul M, West SA, Diggle SP, Griffin AS. (2014). An experimental test of whether cheating is context dependent. J Evol Biol 27: 551–556. 24480013 10.1111/jeb.12319

[bib27] Ghoul M, West SA, McCorkell FA, Lee Z-B, Bruce JB, Griffin AS. (2016). Pyoverdin cheats fail to invade bacterial populations in stationary phase. J Evol Biol 29: 1728–1736. 27223690 10.1111/jeb.12904

[bib28] Ghysels B, Dieu BTM, Beatson SA, Pirnay JP, Ochsner UA, Vasil ML et al. (2004). FpvB, an alternative type I ferripyoverdine receptor of *Pseudomonas aeruginosa*. Microbiology 150: 1671–1680. 15184553 10.1099/mic.0.27035-0

[bib29] Goodman AL, Kulasekara B, Rietsch A, Boyd D, Smith RS, Lory S. (2004). A signaling network reciprocally regulates genes associated with acute infection and chronic persistence in *Pseudomonas aeruginosa*. Dev Cell 7: 745–754. 15525535 10.1016/j.devcel.2004.08.020

[bib30] Granato ET, Harrison F, Kümmerli R, Ross-Gillespie A. (2016). When is a bacterial ‘virulence factor’ really virulent? bioRxiv doi:10.1101/061317. 10.3389/fmicb.2016.01952 PMC5149528 28018298

[bib31] Griffin AS, West SA, Buckling A. (2004). Cooperation and competition in pathogenic bacteria. Nature 430: 1024–1027. 15329720 10.1038/nature02744

[bib32] Hamilton WD. (1964). The genetical evolution of social behaviour I & II. J Theor Biol 7: 1–52. 5875341 10.1016/0022-5193(64)90038-4

[bib33] Harrison F, Browning LE, Vos M, Buckling A. (2006). Cooperation and virulence in acute *Pseudomonas aeruginosa* infections. BMC Biol 4: 21. 16827933 10.1186/1741-7007-4-21 PMC1526758

[bib34] Harrison F, Paul J, Massey RC, Buckling A. (2008). Interspecific competition and siderophore-mediated cooperation in *Pseudomonas aeruginosa*. ISME J 2: 49–55. 18180746 10.1038/ismej.2007.96

[bib35] Harrison F, Buckling A. (2009). Siderophore production and biofilm formation as linked social traits. ISME J 3: 632–634. 19225554 10.1038/ismej.2009.9

[bib36] Harrison F, Buckling A. (2011). Wider access to genotypic space facilitates loss of cooperation in a bacterial mutator. PLoS One 6: e17254. 21364773 10.1371/journal.pone.0017254 PMC3045467

[bib37] Harrison F, Muruli A, Higgins S, Diggle SP. (2014). Development of an *ex vivo* porcine lung model for studying growth Virulence, And signaling of *Pseudomonas aeruginosa*. Infect Immun 82: 3312–3323. 24866798 10.1128/IAI.01554-14 PMC4136229

[bib38] Harrison F, Diggle S. (2016). An *ex vivo* lung model to study bronchioles infected with *Pseudomonas aeruginosa* biofilms. Microbiology. 162: 1755–1760. 27520088 10.1099/mic.0.000352

[bib39] Hirsch EB, Tam VH. (2010). Impact of multidrug-resistant Pseudomonas aeruginosa infection on patient outcomes. Expert Rev Pharmacoecon Outcomes Res 10: 441–451. 20715920 10.1586/erp.10.49 PMC3071543

[bib40] Hohnadel D, Haas D, Meyer JM. (1986). Mapping of mutations affecting pyoverdine production in *Pseudomonas aeruginosa*. FEMS Microbiol Lett 36: 195–199.

[bib41] Hothorn T, Bretz F, Westfall P. (2008). Simultaneous inference in general parametric models. Biom J 50: 346–363. 18481363 10.1002/bimj.200810425

[bib42] Hunt TA, Peng W-T, Loubens I, Storey DG. (2002). The *Pseudomonas aeruginosa* alternative sigma factor PvdS controls exotoxin A expression and is expressed in lung infections associated with cystic fibrosis. Microbiology 148: 3183–3193. 12368452 10.1099/00221287-148-10-3183

[bib43] Hunter RC, Asfour F, Dingemans J, Osuna BL, Samad T, Malfroot A, Cornelis P, Newman DK. (2013). Ferrous iron is a significant component of bioavailable iron in cystic fibrosis airways. mBio 4: e00557–13. 10.1128/mBio.00557-13 PMC3753050 23963183

[bib44] Jiricny N, Diggle SP, West SA, Evans BA, Ballantyne G, Ross-Gillespie A et al. (2010). Fitness correlates with the extent of cheating in a bacterium. J Evol Biol 23: 738–747. 20210835 10.1111/j.1420-9101.2010.01939.x

[bib45] Jiricny N, Molin S, Foster K, Diggle SP, Scanlan PD, Ghoul M et al. (2014). Loss of social behaviours in populations of *Pseudomonas aeruginosa* infecting lungs of patients with cystic fibrosis. PLoS One 9: e83124. 24454693 10.1371/journal.pone.0083124 PMC3891558

[bib46] Koh E-I, Henderson JP. (2015). Microbial Copper-binding siderophores at the host-pathogen interface. J Biol Chem 290: 18967–18974. 26055720 10.1074/jbc.R115.644328 PMC4521018

[bib47] Kirchner S, Fothergill JL, Wright EA, James CE, Mowat E, Winstanley C. (2012). Use of artificial sputum medium to test antibiotic efficacy against *Pseudomonas aeruginosa* in conditions more relevant to the cystic fibrosis lung. J Vis Exp 64: 3857. 10.3791/3857 PMC3471314 22711026

[bib48] Kümmerli R, Gardner A, West SA, Griffin AS. (2009a). Limited dispersal, budding dispersal, and cooperation: an experimental study. Evolution 63: 939–949. 19154373 10.1111/j.1558-5646.2008.00548.x

[bib49] Kümmerli R, Jiricny N, Clarke LS, West SA, Griffin AS. (2009b). Phenotypic plasticity of a cooperative behaviour in bacteria. J Evol Biol 22: 589–598. 19170825 10.1111/j.1420-9101.2008.01666.x

[bib50] Kümmerli R, Griffin AS, West SA, Buckling A, Harrison F. (2009c). Viscous medium promotes cooperation in the pathogenic bacterium *Pseudomonas aeruginosa*. Proc R Soc B Biol Sci 276: 3531–3538. 10.1098/rspb.2009.0861 PMC2817189 19605393

[bib51] Kümmerli R, Brown SP. (2010). Molecular and regulatory properties of a public good shape the evolution of cooperation. Proc Natl Acad Sci USA 107: 18921–18926. 20944065 10.1073/pnas.1011154107 PMC2973908

[bib52] Kümmerli R, Van Den Berg P, Griffin AS, West SA, Gardner A. (2010). Repression of competition favours cooperation: experimental evidence from bacteria. J Evol Biol 23: 699–706. 20487137 10.1111/j.1420-9101.2010.01936.x

[bib53] Kümmerli R, Ross-Gillespie A. (2014). Explaining the sociobiology of pyoverdin producing *Pseudomonas*: a comment on Zhang and Rainey (2013). Evolution 68: 3337–3343. 24219572 10.1111/evo.12311

[bib54] Kümmerli R, Santorelli LA, Granato ET, Dumas Z, Dobay A, Griffin AS et al. (2015). Co-evolutionary dynamics between public good producers and cheats in the bacterium *Pseudomonas aeruginosa*. J Evol Biol 28: 2264–2274. 26348785 10.1111/jeb.12751

[bib55] Leinweber A, Fredrik Inglis R, Kummerli R. (2017). Cheating fosters species co-existence in well-mixed bacterial communities. ISME J 11: 1179–1188. 28060362 10.1038/ismej.2016.195 PMC5437929

[bib56] Lenth RV. (2013), lsmeans: R Package Version 1.06-05.

[bib57] Luján AM, Gómez P, Buckling A. (2015). Siderophore cooperation of the bacterium *Pseudomonas fluorescens* in soil. Biol Lett 11: 20140934. 25694506 10.1098/rsbl.2014.0934 PMC4360104

[bib58] Luong PM, Shogan BD, Zaborin A, Belogortseva N, Shrout JD, Zaborina O et al. (2014). Emergence of the P2 phenotype in *Pseudomonas aeruginosa* PAO1 strains involves various mutations in *MexT* or *MexF*. J Bacteriol 196: 504–513. 24244000 10.1128/JB.01050-13 PMC3911258

[bib59] McCallin K, Cowley E, Reyes MC, Van Sambeek L, Hunter R, Asfour F et al. (2015), Sputum iron levels during cystic fibrosis pulmonary exacerbation: a longitudinal study. American Thoracic Society International Conference Abstracts B52: Pediatric Cystic Fibrosis pp A3343-A3343.

[bib60] Miyazaki H, Kato H, Nakazawa T, Tsuda M. (1995). A positive regulatory gene, *pvdS*, for expression of pyoverdin biosynthetic genes in *Pseudomonas aeruginosa* PAO. Mol Gen Genet MGG 248: 17–24. 7651323 10.1007/BF02456609

[bib61] Morgan AD, Quigley BJZ, Brown SP, Buckling A. (2012). Selection on non-social traits limits the invasion of social cheats. Ecol Lett 15: 841–846 xw. 22639835 10.1111/j.1461-0248.2012.01805.x PMC3444687

[bib62] Mowat E, Paterson S, Fothergill JL, Wright EA, Ledson MJ, Walshaw MJ, Brockhurst MA, Winstanley C. (2011). *Pseudomonas aeruginosa* population diversity and turnover in cystic fibrosis chronic infections. Am J Respir Crit Care Med 183: 1674–1679. 21297072 10.1164/rccm.201009-1430OC

[bib63] Mund A, Diggle SP, Harrison F. (2017). The fitness of *Pseudomonas aeruginosa* quorum sensing signal cheats is influenced by the diffusivity of the environment. mBio 8: e00353–17. 28465424 10.1128/mBio.00353-17 PMC5414003

[bib64] Ochsner UA, Johnson Z, Lamont IL, Cunliffe HE, Vasil ML. (1996). Exotoxin A production in *Pseudomonas aeruginosa* requires the iron-regulated *pvdS* gene encoding an alternative sigma factor. Mol Microbiol 21: 1019–1028. 8885271 10.1046/j.1365-2958.1996.481425.x

[bib65] Palmer KL, Mashburn LM, Singh PK, Whiteley M. (2005). Cystic fibrosis sputum supports growth and cues key aspects of *Pseudomonas aeruginosa* physiology. J Bacteriol 187: 5267–5277. 16030221 10.1128/JB.187.15.5267-5277.2005 PMC1196007

[bib66] Palmer KL, Aye LM, Whiteley M. (2007). Nutritional cues control *Pseudomonas aeruginosa* multicellular behavior in cystic fibrosis sputum. J Bacteriol 189: 8079–8087. 17873029 10.1128/JB.01138-07 PMC2168676

[bib67] Price BL, Lovering AM, Bowling FL, Dobson CB. (2016). Development of a novel collagen wound model to simulate the activity and distribution of antimicrobials in soft tissue during diabetic foot infection. Antimicrob Agents Chemother 60: 6880–6889. 27620475 10.1128/AAC.01064-16 PMC5075099

[bib68] Quinn RA, Lim YW, Maughan H, Conrad D, Rohwer F, Whiteson KL. (2014). Biogeochemical forces shape the composition and physiology of polymicrobial communities in the cystic fibrosis lung. mBio 5: e00956–13. 10.1128/mBio.00956-13 PMC3967525 24643867

[bib69] R Development Core Team (2016)R: A Language and Environment for Statistical Computing. R Foundation for Statistical Computing: Vienna, Austria. Available at: http://www.R-project.org.

[bib70] Rainey PB, Rainey K. (2003). Evolution of cooperation and conflict in experimental bacterial populations. Nature 425: 72–74. 12955142 10.1038/nature01906

[bib71] Raymond B, West SA, Griffin AS, Bonsall MB. (2012). The dynamics of cooperative bacterial virulence in the field. Science 336: 85–88. 10.1126/science.1218196 22767928

[bib72] Rella M, Mercenier A, Haas D. (1985). Transposon insertion mutagenesis of *Pseudomonas aeruginosa* with a Tn5 derivative: application to physical mapping of the *arc* gene cluster. Gene 33: 293–303. 2989092 10.1016/0378-1119(85)90237-9

[bib73] Roberts AEL, Kragh KN, Bjarnsholt T, Diggle SP. (2015). The limitations of *in vitro* experimentation in understanding biofilms and chronic infection. J Mol Biol 427: 3646–3661. 26344834 10.1016/j.jmb.2015.09.002

[bib74] Ross-Gillespie A, Gardner A, West SA, Griffin AS. (2007). Frequency dependence and cooperation: Theory and a test with bacteria. Am Nat 170: 331–342. 17879185 10.1086/519860

[bib75] Ross-Gillespie A, Gardner A, Buckling A, West SA, Griffin AS. (2009). Density dependence and cooperation: theory and a test with bacteria. Evolution 63: 2315–2325. 19453724 10.1111/j.1558-5646.2009.00723.x

[bib76] Ross-Gillespie A, Dumas Z, Kümmerli R. (2015). Evolutionary dynamics of interlinked public goods traits: an experimental study of siderophore production in *Pseudomonas aeruginosa*. J Evol Biol 28: 29–39. 25421271 10.1111/jeb.12559

[bib77] Rumbaugh KP, Diggle SP, Watters CM, Ross-Gillespie A, Griffin AS, West SA. (2009). Quorum sensing and the social evolution of bacterial virulence. Curr Biol 19: 341–345. 19230668 10.1016/j.cub.2009.01.050

[bib78] Sriramulu DD, Lünsdorf H, Lam JS, Römling U. (2005). Microcolony formation: a novel biofilm model of *Pseudomonas aeruginosa* for the cystic fibrosis lung. J Med Microbiol 54: 667–676. 15947432 10.1099/jmm.0.45969-0

[bib79] Stover CK, Pham XQ, Erwin AL, Mizoguchi SD, Warrener P, Hickey MJ et al. (2000). Complete genome sequence of *Pseudomonas aeruginosa* PAO1, an opportunistic pathogen. Nature 406: 959–964. 10984043 10.1038/35023079

[bib80] Székely T, Moore A, Komdeur J. (2010)Social Behaviour: Genes, Ecology and Evolution. Cambridge University Press: Cambridge, UK.

[bib81] Turner KH, Wessel AK, Palmer GC, Murray JL, Whiteley M. (2015). Essential genome of *Pseudomonas aeruginosa* in cystic fibrosis sputum. Proc Natl Acad Sci USA 112: 4110–4115. 25775563 10.1073/pnas.1419677112 PMC4386324

[bib82] Tyrrell J, Callaghan M. (2016). Iron acquisition in the cystic fibrosis lung and potential for novel therapeutic strategies. Microbiology 162: 191–205. 26643057 10.1099/mic.0.000220 PMC4772740

[bib83] Van Opijnen T, Bodi KL, Camilli A. (2009). Tn-seq: high-throughput parallel sequencing for fitness and genetic interaction studies in microorganisms. Nat Methods 6: 767–772. 19767758 10.1038/nmeth.1377 PMC2957483

[bib84] Vasse M, Noble RJ, Akhmetzhanov AR, Torres-Barceló C, Gurney J, Benateau S et al. (2017). Antibiotic stress selects against cooperation in the pathogenic bacterium *Pseudomonas aeruginosa*. Proc Natl Acad Sci 114: 546–551. 28049833 10.1073/pnas.1612522114 PMC5255613

[bib85] Weaver VB, Kolter R. (2004). *Burkholderia* spp. alter *Pseudomonas aeruginosa* physiology through iron sequestration. J Bacteriol 186: 2376–2384. 15060040 10.1128/JB.186.8.2376-2384.2004 PMC412164

[bib86] Werthén M, Henriksson L, Jensen PØ, Sternberg C, Givskov M, Bjarnsholt T. (2010). An *in vitro* model of bacterial infections in wounds and other soft tissues. APMIS 118: 156–164. 20132180 10.1111/j.1600-0463.2009.02580.x

[bib87] West SA, Diggle SP, Buckling A, Gardner A, Griffin AS. (2007). The social lives of microbes. Annu Rev Ecol Evol Syst pp 53–77.

[bib88] Whiteley M, Bangera MG, Bumgarner RE, Parsek MR, Teitzel GM, Lory S et al. (2001). Gene expression in *Pseudomonas aeruginosa* biofilms. Nature 413: 860–864. 11677611 10.1038/35101627

[bib89] Wilson MJ, McMorran BJ, Lamont IL. (2001). Analysis of promoters recognized by PvdS, an extracytoplasmic-function sigma factor protein from *Pseudomonas aeruginosa*. J Bacteriol 183: 2151–2155. 11222621 10.1128/JB.183.6.2151-2155.2001 PMC95118

[bib90] Yang L, Jelsbak L, Marvig RL, Damkiær S, Workman CT, Rau MH et al. (2011). Evolutionary dynamics of bacteria in a human host environment. Proc Natl Acad Sci USA 108: 7481–7486. 21518885 10.1073/pnas.1018249108 PMC3088582

[bib91] Zhang X-X, Rainey PB. (2013). Explaining the sociobiology of pyoverdin producing Pseudomonas. Evolution 67: 3161–3174. 24152000 10.1111/evo.12183

