# Supplementary material for: Optimised chronic infection models demonstrate that siderophore ‘cheating’ in Pseudomonas aeruginosa is context specific
Source: ISME J. 2017 Jul 11;11(11):2492–509. doi: 10.1038/ismej.2017.103 (PMC5649161; doi:10.1038/ismej.2017.103)
Supplement: Supplementary Figures [file ismej2017103x1.docx]

**Figure S1.** The genealogy of PA06609 (a.k.a. PAO9), as reconstructed from the literature.

**
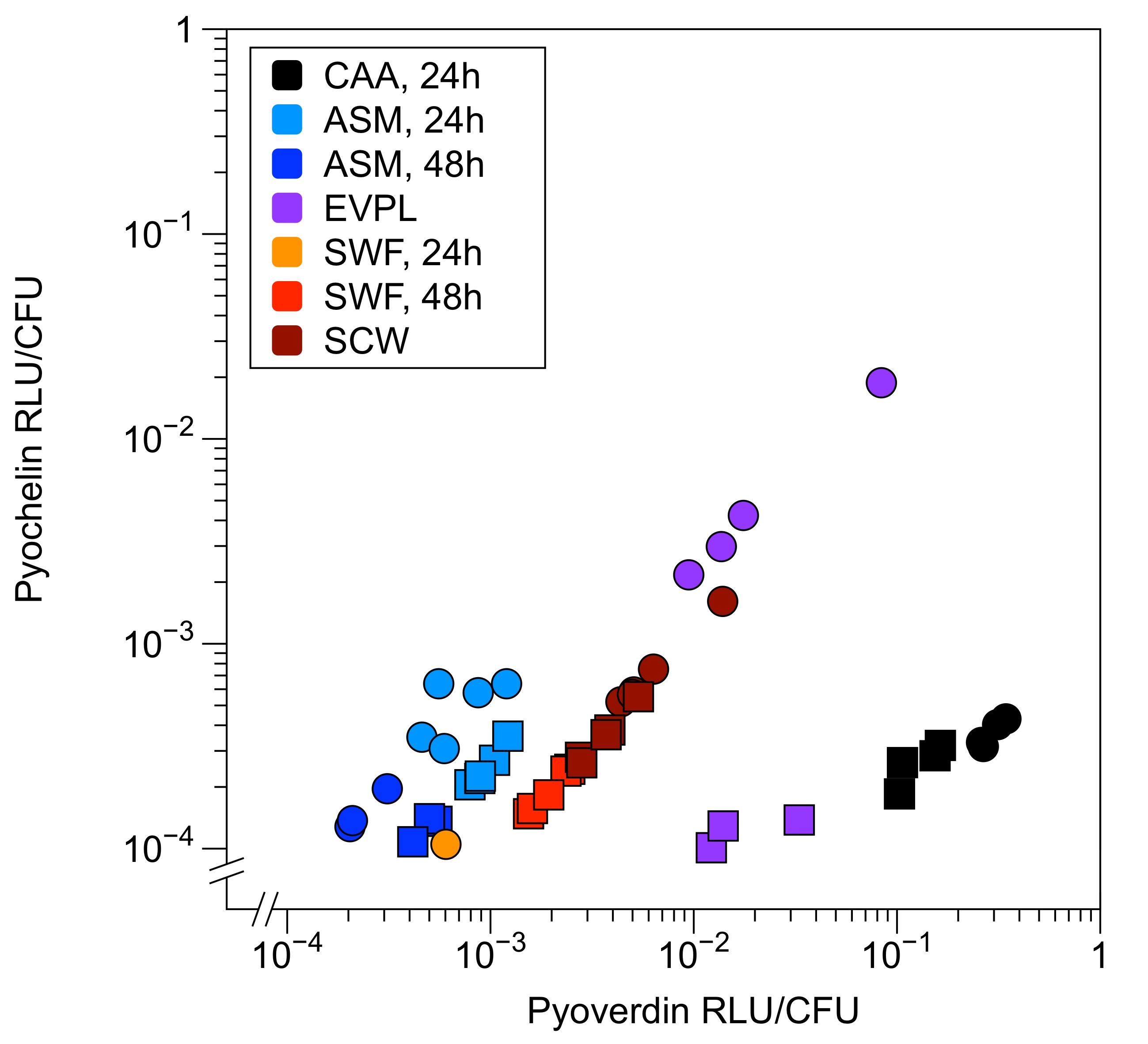
**

**
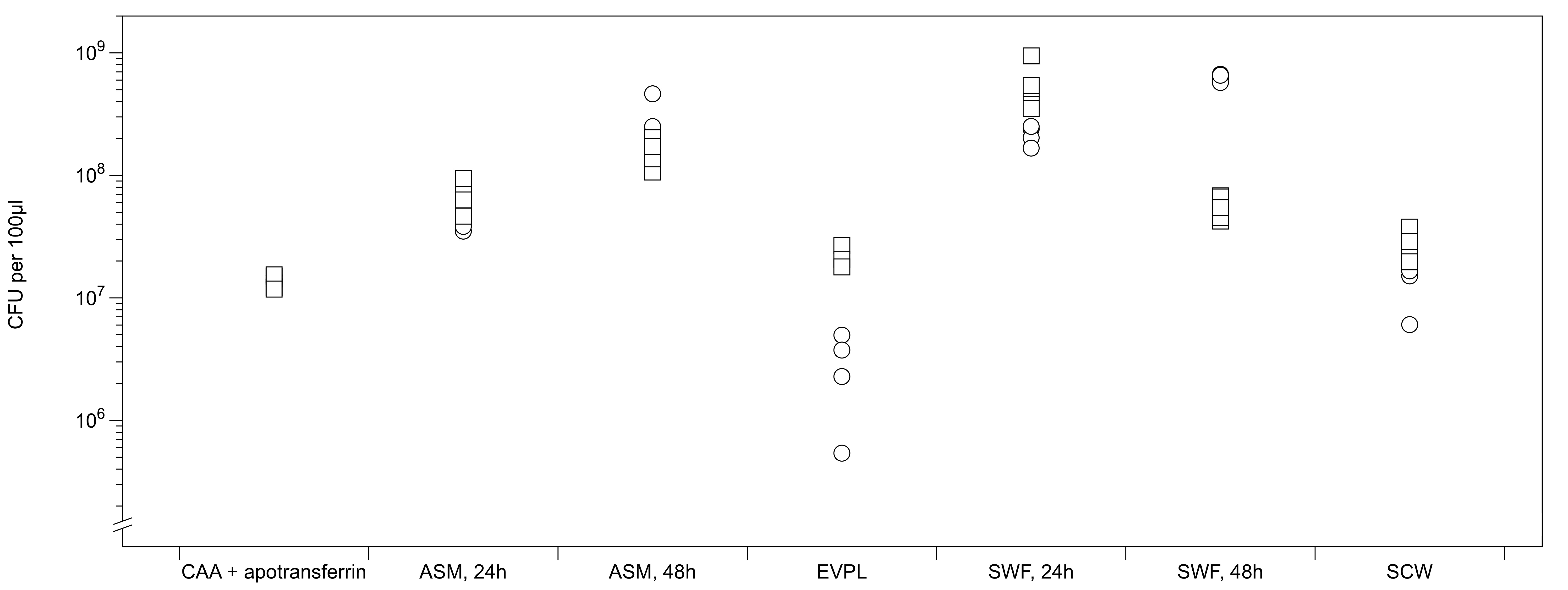
**

**Figure S2.** (a) Levels of pyoverdin and pyochelin produced by wild-type *P. aeruginosa* in the different environments explored in this study. Molecules were detected by excitation-emission assays of 100 µl aliquots of culture supernatant and expressed as relative luminescence units (RLU) divided by the number of *P. aeruginosa* colony-forming units (CFU) present in 100 µl of the original culture. Symbols denote experimental block. (b) Bacterial density (CFU per 100 µl) the different environments. Symbols denote experimental block. CAA: casamino acids medium, ASM: artificial sputum medium, EVPL: *ex vivo* pig lung model, SWF: synthetic wound fluid, SCW: synthetic chronic wound model.


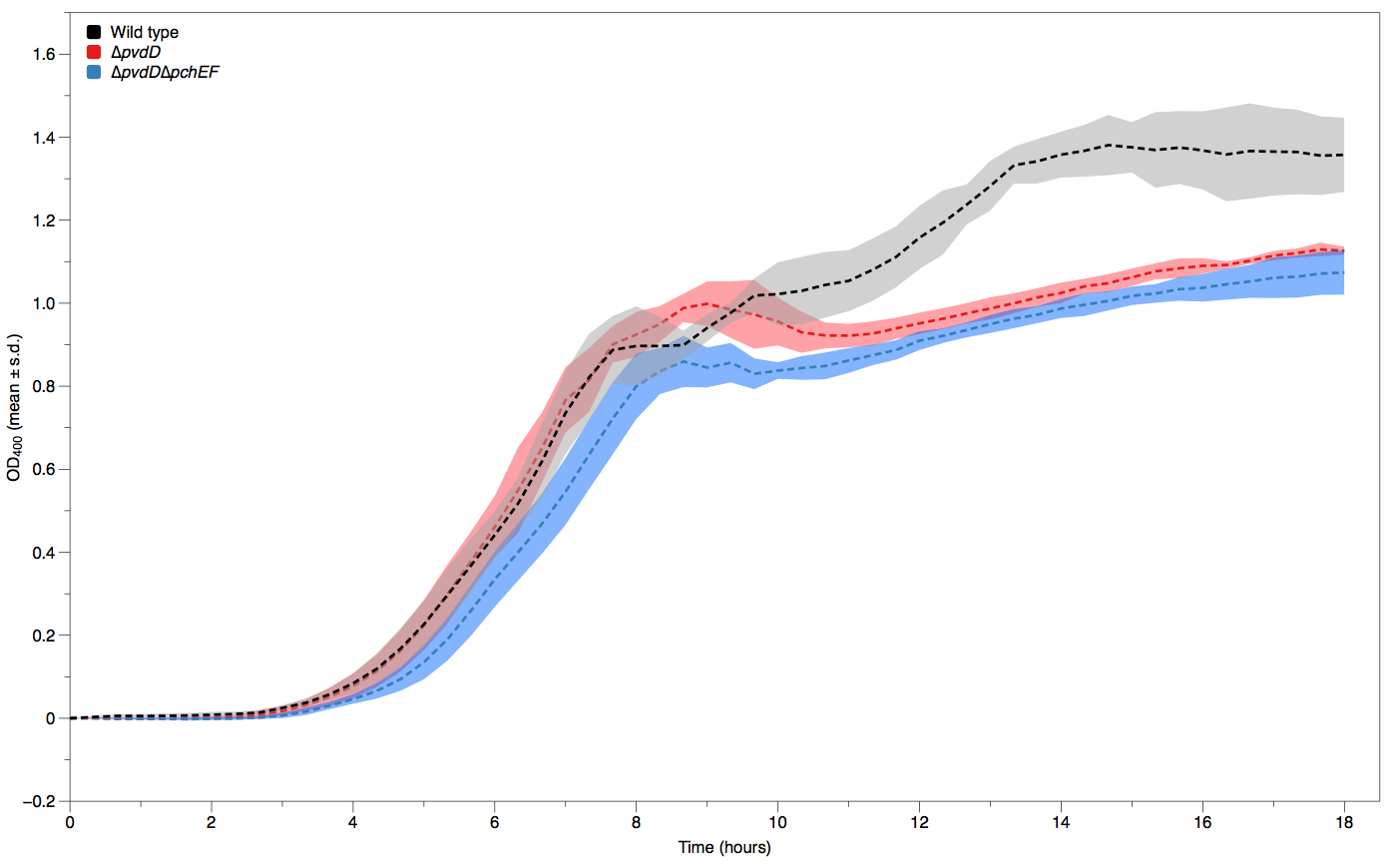


**Figure S3.** Growth of wild type (black), ∆pvdD (red) and ∆pvdD∆pchEF (blue) *P. aeruginosa* in 200 µl ASM over 18 hours. Optical density was read at 400 nm to minimise interference from pyoverdine absorbance in wild-type cultures. Lines show means of four replica cultures, shaded areas show ± one standard deviation.
